# Exploring the Use of Podcasting in the Medical Radiation Sciences

**DOI:** 10.1002/hsr2.72237

**Published:** 2026-04-02

**Authors:** Elio Arruzza, Bismark Ofori‐Manteaw, Don Nocum, Tarni Nelson, Dania Abu Awwad, Jacob Leonard Ago, Minh Chau, Tooba Zaidi, Yobelli Jimenez

**Affiliations:** ^1^ School of Allied Health & Human Performance Adelaide University Adelaide South Australia Australia; ^2^ Teaching in Radiography Group, Faculty of Science and Health Charles Sturt University Wagga Wagga New South Wales; ^3^ Faculty of Science and Health Charles Sturt University Wagga Wagga New South Wales Australia; ^4^ Discipline of Medical Imaging Science, Faculty of Medicine and Health The University of Sydney Camperdown New South Wales Australia; ^5^ Discipline of Medical Radiations, School of Health and Biomedical Sciences RMIT University, Bundoora Campus Victoria Australia; ^6^ College of Health Sciences VinUniversity Ha Noi Vietnam

**Keywords:** education, nuclear medicine, podcasts, radiation therapy, radiography

## Abstract

**Background and Aims:**

The integration of digital media into health professions education has reshaped how knowledge is disseminated, accessed, and applied in both clinical and academic settings. This review explored the published literature relating to podcasting in the Medical Radiation Sciences (MRS) and synthesized the scope of publicly available MRS‐related podcasts.

**Methods:**

A two‐part methodology was used. Firstly, Apple Podcasts and Spotify were searched using relevant terms, and data were extracted on topic content, structure, target audience, and format. Secondly, a review of published journal articles was conducted using major literature databases.

**Results:**

Of the 280 podcasts screened, 25 met the inclusion criteria. These included podcasts dedicated to medical imaging (*n* = 9), radiation therapy (*n* = 8), both disciplines (*n* = 4), nuclear medicine (*n* = 2), and all three (*n* = 2). They originated from the USA (*n* = 13), UK (*n* = 6), Australia (*n* = 4), Canada (*n* = 1), and Ireland (*n* = 1), with episode counts ranging from 2 to 427. Four journal articles were identified, reporting on podcasting as a strategy to enhance student engagement, support well‐being, and explore challenging clinical topics. Informal formats, co‐design with students, and integration into curricula were common.

**Conclusion:**

While anecdotal feedback and engagement data were positive, few studies used formal outcome measures. Podcasting shows promise as a flexible, learner‐centred tool in MRS education.

## 1 Introduction

In recent years, the integration of digital media into health professions education has reshaped how knowledge is disseminated, accessed, and applied in clinical and academic settings. Within the Medical Radiation Sciences (MRS), social and digital media platforms have increasingly been used to support education, professional engagement, and communication, reflecting broader shifts towards technology‐enhanced learning [1]. Among the diverse range of digital tools available, podcasts have gained increasing prominence as an informal yet powerful medium for delivering educational content [2]. Characterized by their portability, flexibility, and ease of access, podcasts offer a learner‐centred alternative to traditional instructional methods, enabling professionals and students to engage with content on‐demand and in diverse contexts, whether during commutes, clinical breaks, or personal study time [3, 4, 5, 6, 7].

This shift towards flexible, technology‐enhanced learning is not merely a response to changing learner preferences; it also reflects broader transformations in health education aimed at fostering lifelong learning, interdisciplinary engagement, and just‐in‐time knowledge acquisition [2, 8]. As a result, many disciplines have embraced podcasts as a supplement to formal instruction and a means of promoting continuous professional development [8, 9]. Podcasts are episodic audio programs distributed via the internet, typically accessible through dedicated platforms such as Apple Podcasts and Spotify [10]. They are designed for asynchronous use, allowing users to download or stream content at their convenience [4, 7]. In the context of health professions education, podcasts have been increasingly recognized as a versatile tool for delivering clinical updates, reinforcing theoretical concepts, sharing expert insights, and exploring emerging trends in healthcare practice [11, 12].

The pedagogical value of podcasts lies in their ability to support self‐directed learning, promote knowledge retention through audio engagement, and accommodate diverse learning styles [13, 14, 15]. They enable content delivery beyond the constraints of traditional classroom settings and provide learners with opportunities to revisit material as needed [4]. Podcasts are also often produced by clinicians, educators, or professional bodies, which enhances their relevance and credibility within specific health disciplines [16, 17, 18]. Several studies have demonstrated the efficacy of podcasts in enhancing learner motivation, satisfaction, engagement, and perceived competence [3, 11, 16, 18]. These outcomes have positioned podcasts not only as supplementary learning tools but also as accessible resources for professional development, especially in contexts where time, geography, or access to formal learning opportunities may pose constraints [16].

Podcasts present a promising medium for addressing these needs within the MRS discipline. They offer the potential to complement formal education by reinforcing foundational knowledge, sharing real‐world clinical experiences, and disseminating professional insights in an engaging, time‐efficient manner [19, 20]. For students, podcasts can enhance understanding of core concepts and bridge the theory–practice gap [7, 13, 19]. For practitioners, they offer opportunities for reflective learning and professional development that align with busy clinical schedules [17]. According to Casares [6], podcasts are as beneficial as reading an academic journal article to promote and improve healthcare professionals' evidence‐based practice.

Despite these documented benefits, the adoption and integration of podcasts within the MRS disciplines remain underexplored. There is a paucity of research that maps the availability, scope, and content of publicly accessible podcasts specifically tailored to the MRS context. In a systematic review, Clarke et al. [21] identified forty‐one podcasts related to radiology. Of the three podcasts identified as “radiography”, only one was active at the time of the review [21]. As a result, it remains unclear which topics are being addressed, who the primary content creators and target audiences are, and how consistently new content is being produced and disseminated. More so, the academic literature investigating the development, implementation, and pedagogical effectiveness of podcasts in MRS education and practice is limited.

Unlike other health professions where podcasts have become a mainstream educational resource, the extent to which MRS educators, students, and clinicians utilize or benefit from podcast content is unclear. This lack of empirical evidence limits our understanding of the extent to which podcasts are being used, valued, or evaluated in MRS. This review is therefore timely, aiming to bridge this gap by examining both publicly available podcast content and the academic literature related to podcast use in MRS. By mapping the current podcast landscape and synthesizing existing evidence, the review aims to identify key themes, gaps, and opportunities for enhancing education and professional development through podcasting in MRS.

## 2 Methods

This review underwent a two‐part approach. The first part involved a content analysis to review and characterize eligible podcasts relating to MRS on Apple Podcasts (Apple Inc., Cupertino, CA) and Spotify (Spotify AB, Stockholm, Sweden). The research involved publicly available data (podcasts) and did not involve data collection from human subjects, so ethical approval was not required.

The review was conducted on the Apple Podcasts (Australia) and Spotify (Australia) applications in June 2025. The keywords “radiography”, “radiation therapy”, “radiotherapy”, “diagnostic radiography”, “nuclear medicine”, “medical radiation”, and “medical imaging” were each searched in each application's search directory. Following this, the first 20 results for each keyword were reviewed as part of this study, due to the decreasing relevance of podcasts beyond those results, resulting in 280 podcast series reviewed in total. Following exclusion of duplicates, each podcast show's title and description were evaluated for inclusion. Podcasts were excluded if they: (1) were not related to the scope of practice associated with diagnostic radiographers, radiation therapists, and nuclear medicine technologists, (2) did not have at least one episode, or (3) the content was not in English. Once a list of podcasts meeting the inclusion criteria was generated, data were extracted from the podcast description and episode list into a Microsoft Excel spreadsheet (Microsoft Corporation, United States). The data extracted included: podcast name, authors, country of origin, number of episodes, date of first and last episode, duration (shortest and longest episode length), discipline (i.e., medical imaging, radiation therapy, nuclear medicine), format (audio, video), and whether it contained supplemental information. Quantitative content analyses were performed, with frequency of content expressed as percentages within categories. This was undertaken to both summarize and report on details concerning the included podcasts.

The second part involved a literature review to identify and evaluate published research related to the use of podcasts within the field of MRS. The databases searched included Medline, Scopus, and CINAHL, covering journal articles published up to June 2025. The search strategy was as follows: “podcast*” AND *(“*medical radiation sciences” OR “radiography” OR “radiation therapy” OR “nuclear medicine” OR “medical imaging”). Inclusion criteria were any full‐text published English language literature addressing podcasts as an educational or professional development tool within the MRS disciplines. Exclusion criteria included articles that focused on podcast use in broader medical or allied health education without specific reference to MRS. Titles and abstracts were screened for relevance, followed by full‐text reviews of eligible articles. Key data were extracted from each study, including publication year, country of origin, study design, sample size and population, podcast topic and format, and reported outcomes or findings. The extracted data were synthesized to identify common themes, educational applications, and research gaps related to podcast use in MRS education and practice.

## 3 Results

### 3.1 Part I

In total, 25 podcast series met the inclusion criteria for MRS‐specific podcasts. Table 1 summarizes the key characteristics of available podcasts. Identified podcast shows related to medical imaging (*n* = 9), radiation therapy (*n* = 8), nuclear medicine (*n* = 2), both medical imaging and radiation therapy (*n* = 4), or medical imaging, radiation therapy, and nuclear medicine (*n* = 2). They originated from the United States of America (USA) (*n* = 13), the United Kingdom (UK) (*n* = 6), Australia (*n* = 4), Canada (*n* = 1), and Ireland (*n* = 1). The number of episodes for each podcast ranged from 2 to 427. The podcast series “RadChat” had the largest number of published episodes (*n* = 427), beginning in 2019. Most podcast series were audio only (*n* = 20, 80%), compared to the inclusion of video (*n* = 5, 20%). The majority of the podcasts (*n* = 21) were active at the time of data collection, defined in this review as having new episodes released within the previous 6 months to the date of data collection. Many of the podcasts were educational in nature, featuring interviews with industry experts, and discussing trends, research, and clinical practice. Three of the podcasts were associated with MRS‐based journals, whilst another two were affiliated with MRS societies. A comedy podcast was also discovered.

**Table 1:** Characteristics of MRS‐specific podcasts as found on the Apple Podcasts and Spotify platforms.

Podcast name (authorship)	Country of origin	Number of episodes	Duration in minutes (min, max) episode length	Year of first and latest episode	Main discipline	Summary of topic (content areas)	Format	
							Audio	Video
A couple of rad techs podcasts (Chaundria)	USA	96	2, 84	2021, 2025	MI, RT	A married couple of radiation technologists explore the latest techniques, technologies, and innovations in medical imaging.	✓	
Advanced practice radiation therapy international community of practice podcast series	USA	5	21, 90	2023, 2025	RT	Knowledge exchange, dissemination of new RT techniques, international community of practice.	✓	✓
All about radiotherapy (CRUK Cambridge centre)	UK	16	5,30	2022, 2023	RT	Discussion with professionals critical for the delivery of radiotherapy treatment today, and conversations about the history of radiotherapy since the 1970s.	✓	
Breathe In… radiography podcast (Emily Girard)	AUS	9	4, 31	2021, 2022	MI	Podcast to support diagnostic radiography students, focused on student wellbeing and placements.	✓	
Catheter jockeys: a rad tech podcast (Catheter jockeys production)	USA	214	32, 72	2022, 2024	MI	Comedy podcast discussing the experiences of the hosts within the medical imaging industry.	✓	
Everything MRI podcast (everything MRI)	UK	15	43, 62	2023, 2025	MI	Expert hosts and guest interviews, featuring topics ranging from “remote scanning, to artificial intelligence (A.I.) implementation, patient positioning, and physics and much more”.	✓	✓
JNM (journal of nuclear medicine) podcast	USA	10	21, 43	2024, 2025	NM	Official podcast of the journal of nuclear medicine (JNM), featuring editors and guest experts to discuss emerging technologies and rapidly changing issues in practice and research.	✓	
Medical imaging matters	USA	12	14, 41	2018, 2025	MI	Latest industry trends and practices that matter to medical imaging leaders.	✓	
Out of the Gray (Gy) standard imaging (Traci Conley)	USA	97	12, 56	2021, 2025	RT	Expert interviews discussing new ideas, research, and the future of radiation oncology technology.	✓	
Patient experience of radiation therapy (Michelle Leech)	IRL	2	11, 26	2025	RT	Interviews with patients as they share. their challenges and insights in receiving radiation therapy.	✓	
Professional button pushers (Tarni Nelson)	AUS	16	26, 54	2021, 2025	MI, NM, RT	Interviews industry clinicians for insight into the profession for undergraduate medical radiation science students.	✓	
Rad chat (Naman Julka‐Anderson and Jo McNamara)	UK	427	3, 57	2019, 2025	RT	Podcast sharing real‐world experience, expert insights, best practices, and patient perspectives, to advance cancer care and improve patient outcomes.	✓	
Radiation station (Burlington medical)	USA	6	30, 75	2025	RT, MI, NM	Discussions focused on radiation protection.	✓	
Radiation therapist: beaming with confidence! (Manya Betageri)	USA	20	4, 38	2024, 2025	RT	Essential radiation therapy curriculum content.	✓	
Radiography revision (bonniecaitlin12‐au)	USA	5	14, 22	2025	MI	A new podcast used for radiography revision on x‐ray protection, emission, and tube.	✓	
Radiology roundup (Melanie Hail)	USA	11	21, 87	2025	MI	Conversations with experts regarding radiologic technology and healthcare education.	✓	✓
Radiotherapy and its physics (The Open University)	UK	52	2, 7	2008, 2010	RT	Masters level science course to teach application of physics to the techniques of radiation therapy.	✓	✓
RadPod ASMIRT (Australian society of medical imaging and radiation therapy)	AUS	2	16, 23	2025	MI, RT	The official podcast of the Australian society of medical imaging and radiation therapy (ASMIRT), featuring expert interviews for professionals and students.	✓	
Spilling the RT (Elekta)	UK	4	30, 47	2025	RT	Conversations with experts in radiation therapy.	✓	
Study pathology for radiography (Cady Johnson)	USA	9	8, 41	2025	MI	Uses artificial intelligence to generate notes into a podcast for pathology in a radiography program.	✓	
The journal of medical imaging and radiation sciences (the journal of medical imaging and radiation sciences)	CAN	19	9, 50	2020, 2025	MI, RT	A regular podcast associated with the journal of medical imaging and radiation science (JMIRS), featuring expert interviews and discussion	✓	
The nuclear medicine and molecular medicine podcast (Rob Williams)	AUS	25	9, 40	2022, 2025	NM	Discussion of the latest practices for NM and positron emission tomography (PET) professionals, with expert interviews.	✓	✓
The student radiographer podcast (society of radiographers student network)	UK	6	31, 64	2024, 2025	MI, RT	A podcast “for student radiographers, by student radiographers”.	✓	
Your x‐ray tech podcast (Jason Hernandez)	USA	39	1, 52	2022, 2025	MI	Host and expert interviews, with topics ranging from the basics of medical imaging to the latest advancements in the field.	✓	
5 min radiography (Jeremy Infringe**r**)	USA	16	3, 20	2019, 2025	MI	Short, host‐led educational recordings with emphasis on patient care, technological changes, and ALARA (as low as reasonably achievable).	✓	

Abbreviations: AUS, Australia; CAN, Canada; IRL, Ireland; MI, medical imaging; NM, nuclear medicine; RT, radiation therapy; UK, United Kingdom; USA, United States of America.

### 3.2 Part II

Four published studies were discovered in the literature review, with the earliest mention being in 2009. A summary of these studies is found in Table 2, all of which pertain to diagnostic radiography specifically. While podcasting was still in its infancy, Lorimer and Hilliard recognized its potential as part of a suite of tools to support flexible, learner‐centred education [22]. The paper emphasized the importance of embracing new modalities to supplement traditional teaching and highlighted both the enthusiasm and hesitancy among educators to adopt such innovations. Nelson and Bilton piloted an extracurricular podcast targeted at undergraduate MRS students, aiming to increase engagement, professional awareness, and self‐directed learning [12]. The podcast episodes included discussions on current clinical issues, interviews with professionals, and reflections on the student experience. Preliminary data were gathered through listener analytics and anecdotal feedback, which indicated positive student engagement and high levels of reach across regions and devices [12]. The study positioned podcasting as a novel, low‐cost strategy to complement formal curricula, particularly valuable in remote or asynchronous learning environments. The most recent two papers by Girard and colleagues reported on the development and evaluation of a wellbeing‐focused podcast titled “Breathe in Radiography” which aimed to support students during their clinical placements [10, 23]. Episodes explored themes such as burnout, self‐care, and managing difficult situations. Listener analytics indicated steady engagement, though formal outcome evaluations (e.g., using psychological measures) were not included.

**Table 2:** Literature review of podcasting in medical radiation sciences.

Author and year	Journal	Country	Article/year	Discipline	Associated podcast
Lorimer and Hilliard (2009) [22]	Radiography	UK	Incorporating learning technologies into undergraduate radiography education	MI	Not reported
Nelson and Bilton (2021) [12]	Journal of Medical Imaging and Radiation Sciences	AUS	Podcasting to the undergraduate medical radiation student (2021)	MI, RT, NM	Professional button pushers (Tarni Nelson)
Girard, Punch, and Jimenez (2024) [23]	Journal of Medical Radiation Sciences	AUS	Framework for a radiography student podcast (2024a)	MI	Breathe in radiography (Emily Girard)
Girard, Punch, and Jimenez (2024) [10]	Journal of Medical Radiation Sciences	AUS	A wellbeing podcast for diagnostic radiography students (2024b)	MI	Breathe in radiography (Emily Girard)

## 4 Discussion

This review aimed to summarize the current landscape of podcasts within MRS. The contemporary nature of podcasting in the field is highlighted by the recent availability of podcasts on the Apple Podcasts and Spotify platforms, as well as in published literature. It was evident that the availability of MRS podcasts is growing, with 88% (*n* = 22) of included podcasts in this review publishing their first episode since the year 2020. This growing trend is in line with similar reports in health and other sectors [24, 25]. As a greater number of MRS podcasts become available, greater uptake may in turn increase the demand and potential for further expansion of MRS‐related podcasts in the future.

The evolving landscape of MRS professions demands ongoing engagement with new knowledge, evolving technologies, and interdisciplinary collaboration. As healthcare delivery becomes increasingly complex, MRS professionals are expected to stay abreast of current evidence, best practices, and innovations across clinical, educational, and regulatory domains. This need for continuous learning has prompted a growing interest in accessible, flexible, and contextually relevant educational tools.

The wide‐ranging content in the included podcasts, summarized in Table 1 reflects current issues in MRS. Much of the content contained within the included podcasts entailed a dissemination and conversation about research findings, new techniques, and technological advancement. These podcasts help to further promote professionals' engagement with research evidence and may serve as a continuing professional development tool. Within this context, podcasts have the potential to provide information in a less intrusive and formal way compared to traditional teaching opportunities [16, 26].

Given that only four published studies were found, this indicates limited evidence demonstrating the integration of podcasts into MRS education. Nevertheless, a key takeaway of the published studies was their emphasis on co‐design and student agency, involving students directly in topic selection and content creation [10, 12, 23]. These approaches align with learner‐centred pedagogy and increase relevance and engagement. Implementation styles varied, ranging from informal, extracurricular models to curriculum‐integrated approaches. This illustrates the versatility of podcasting as a medium. Collectively, these studies demonstrate that when thoughtfully developed and aligned with learner needs, podcasts can be a flexible and meaningful tool to support both the academic and emotional aspects of student learning.

From a practical perspective, our findings suggest several potential applications for podcasting within MRS education. Podcasts may be incorporated into undergraduate and postgraduate curricula as supplementary learning resources, particularly within blended or flipped learning models. For practising professionals, podcasts offer a flexible CPD modality that can be accessed during commuting or downtime, potentially reducing barriers related to time and workload.

While the reviewed studies highlight the promise of podcasting as an educational tool in MRS, a notable limitation across the literature is the lack of rigorous evaluation. Most studies relied on descriptive feedback, usage analytics, or anecdotal commentary to assess impact, with limited use of validated tools or formal assessment measures. No research has employed pre‐ and post‐intervention testing, control groups, or longitudinal follow‐up, making it difficult to determine the effectiveness of podcasting in the MRS context. Furthermore, there is an absence of research into how podcasting influences clinical performance, resilience, or well‐being. Future research should prioritize robust study designs to evaluate the pedagogical and psychological outcomes of podcast use, including mixed‐methods approaches that capture both quantitative and qualitative data. In addition, expanding research beyond diagnostic radiography to include radiation therapy, nuclear medicine, and interprofessional contexts would provide a more comprehensive understanding of podcasting's role across the full spectrum of MRS education.

This review has several limitations that should be acknowledged. First, the podcast search was conducted on only two podcast streaming platforms (Apple Podcasts and Spotify). Although these platforms are widely used within the Australian context and host a large proportion of health‐related podcasts, it is possible that relevant podcasts are available on other directories. Additionally, platform‐specific indexing may not have encompassed all episodes within a podcast series. For example, while only 25 episodes since 2022 of *The Nuclear Medicine and Molecular Medicine Podcast* were identified, further investigation revealed that the podcast commenced in 2005. Second, the search strategy was limited to the first 20 results per keyword. This decision was made after determining that results beyond this point demonstrated a marked decline in relevance to medical radiation science. Nevertheless, this approach may have limited the identification of less prominent or emerging podcasts. Third, the review was limited to podcasts published in English, which may have excluded relevant non‐English content and reduced the international scope of the findings. Finally, this study was designed as a narrative literature review and did not follow formal systematic review methodology. While appropriate for the exploratory aims of this review, this methodological approach may affect reproducibility and increase the risk of selection bias.

## 5 Conclusion

The findings of this review support the interests of the MRS community in creating and engaging with podcasts relevant to their profession. It also serves to identify gaps in topics and challenges faced by the professional community that can be addressed through a flexible and entertaining educational method. The analysis of the identified podcasts and podcast‐related peer‐reviewed literature shows promising potential in MRS education and professional development. Of note, many of the MRS podcasts were produced by credible professionals and organizations. This review identified that existing podcasts tend to focus on clinical updates, professional experiences, and research dissemination. Podcasts also hold potential for supporting student learning when co‐designed with learners and aligned with their needs. There is therefore a need for the MRS educators, researchers, and professional groups to develop podcasts that address the identified gaps in clinical education and support reflective learning. Furthermore, future studies should adopt rigorous designs, such as pre‐test/post‐test study designs, to evaluate the utility and effectiveness of podcasts. A well‐designed podcast may greatly enhance academic and clinical education and support lifelong learning across the MRS profession.

## Author Contributions

**Elio Arruzza:** writing – review and editing, writing – original draft, conceptualization, formal analysis, methodology, and investigation. **Bismark Ofori‐Manteaw:** writing – original draft, writing – review and editing, and investigation. **Don Nocum:** formal analysis, investigation, data curation, writing – original draft, writing – review and editing. **Tarni Nelson:** writing – original draft, writing – review and editing, formal analysis, and investigation. **Dania Abu Awwad:** formal analysis, investigation, data curation, writing – original draft, writing – review and editing. **Jacob Leonard Ago:** investigation, writing – original draft, writing – review and editing. **Minh Chau:** writing – original draft, writing – review and editing, and investigation. **Tooba Zaidi:** investigation, writing – original draft, writing – review and editing, and formal analysis. **Yobelli Jimenez:** conceptualization, methodology, formal analysis, investigation, data curation, writing – original draft, writing – review and editing, and project administration.

## Funding

The authors have nothing to report.

## Conflicts of Interest

The authors declare no conflicts of interest.

## Transparency Statement

The lead author, Elio Arruzza, affirms that this manuscript is an honest, accurate, and transparent account of the study being reported; that no important aspects of the study have been omitted; and that any discrepancies from the study as planned (and, if relevant, registered) have been explained.

## Data Availability

The authors confirm that the data supporting the findings of this study are available within the article.
